# The dichotomy of diagnostics: exploring the value for consumers, clinicians and care pathways

**DOI:** 10.1038/s41746-024-01087-8

**Published:** 2024-04-23

**Authors:** Dylan Powell, Aiden Hannah

**Affiliations:** 1 Faculty of Health Sciences & Sport, University of Stirling, Stirling, UK; 2 Glasgow, UK

## Abstract

Diagnostics play a crucial role in screening, detecting, and stratifying patients, yet can account for only 2–3% of healthcare spending. With advancements in wearable technology and direct-to-consumer testing, the market for consumer health continues to rise. The potential benefits of more holistic and continuous measurement offer a promising opportunity for earlier disease detection and proactive health management. Many health systems are in a parallel transition from legacy analogue approaches to digitally enabled infrastructures. The evolving role of the clinical workforce, including medical ethics, regulation, will be closely coupled and a critical lever in success. This includes on a patient and clinician level, balancing the benefits and risks of interventions, and care pathway level, promoting responsible data utilisation with greater contextualisation based on the latest evidence of clinical efficacy. Moving forward a balance may need to be struck between increased data capture, analysis and reuse, with proportionate ethics, regulation, trust and governance.

## The case for change

Healthcare systems globally face significant and growing challenges, from climate change’s significant threat to human health, global workforce shortages and supply chain challenges, to declining life expectancy and growing inequalities^1,2^. All whilst the cost of healthcare increases in many countries. At the heart of responding to these challenges is the need for an integrated approach to innovation and service redesign to ensure that healthcare systems are fit for purpose, are sustainable, and deliver a positive impact for clinicians, consumers, and communities. Embracing innovation and embedding technology throughout the healthcare ecosystem will be critical to tackling these issues head-on. However adapting and shaping the wider approach to healthcare will also be key, including how regulation, workforce and medical ethics are ‘drivers not passengers’ in this change.

## The crucial role of diagnostics

Diagnostic technologies play a pivotal role across the care continuum in screening, detecting and stratifying patients^1,3^. Within the NHS, it has been estimated over 85% of patients seeking care will require some form of diagnostic technology^4^. Despite influencing 70 per cent of medical decision-making, diagnostics can account for in some circumstances only 2–3 per cent of healthcare spending^5^. In the ever-evolving healthcare landscape, diagnostics occupies a curious position^2,6,7^. They hold the potential power to illuminate unseen dangers, guide personalised treatment to unlock precision medicine, with early diagnosis a key tool in improving patient outcomes and reducing demand on downstream primary and secondary care services^8–10^. Moreover, diagnostics and assessments are no longer bound to clinics or laboratories, and can now take place ‘where the patient is’ (often at home or work) instead of where the clinician is^11^. Additionally, an increased availability of technologies has facilitated some individuals to become more self-aware of their own health status in a connected and data-driven manner^2,9^.

**Fig. 1:**
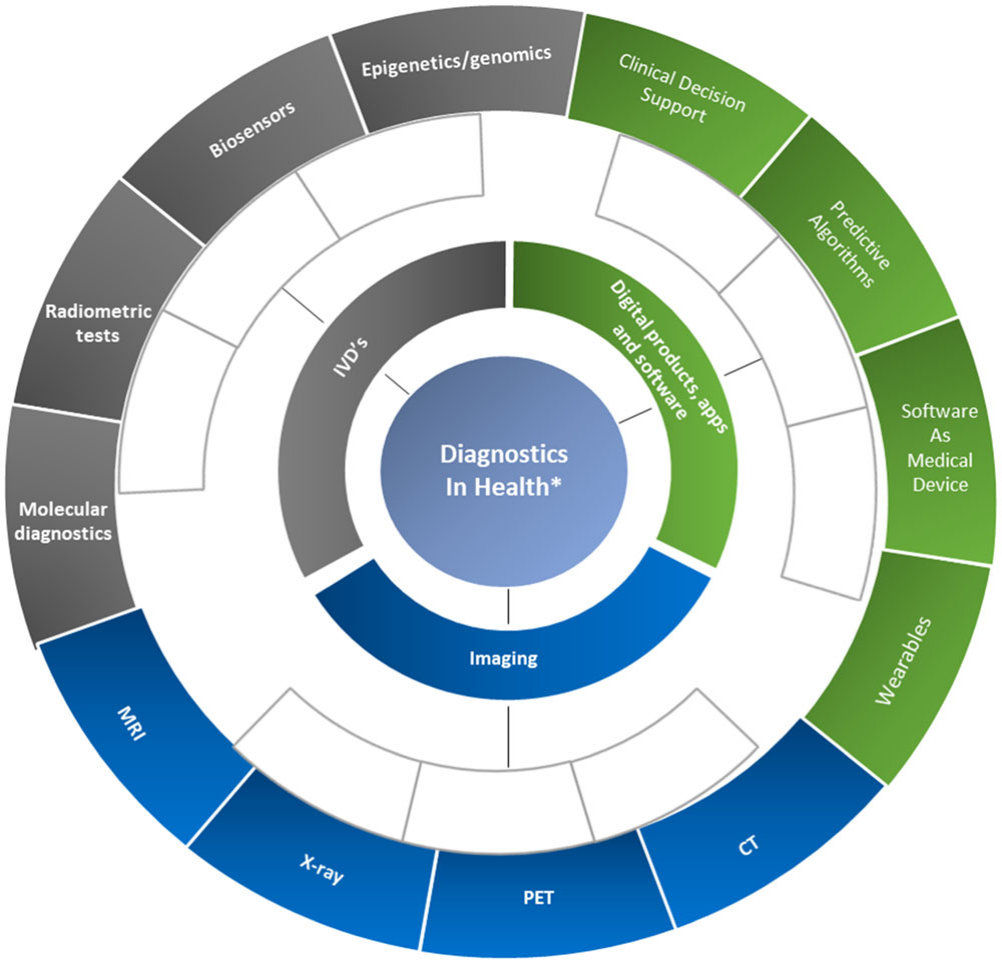
Scope and range of diagnostic approaches within health. Parts of this figure utilises photos from Stirling University Brand Bank (https://www.stir.ac.uk/brand-bank/visual-assets/) all licensed under CC BY-SA.

This paradigm shift may also have broader value and power through keeping individuals ‘well’ through primary prevention and delaying morbidity. However, as recently outlined, with great power ‘comes great responsibility’, and the unforeseen impacts of the perceived positive quantified self also need to be evaluated, including the evidence base for monitoring, and how data will be gathered, used, and re-used^12^. This increasingly is blurring the boundaries of diagnostics and consumer health^12,13^ (Fig. 1).

## The intertwined challenges of diagnostics and consumer health data

**Fig. 2:**
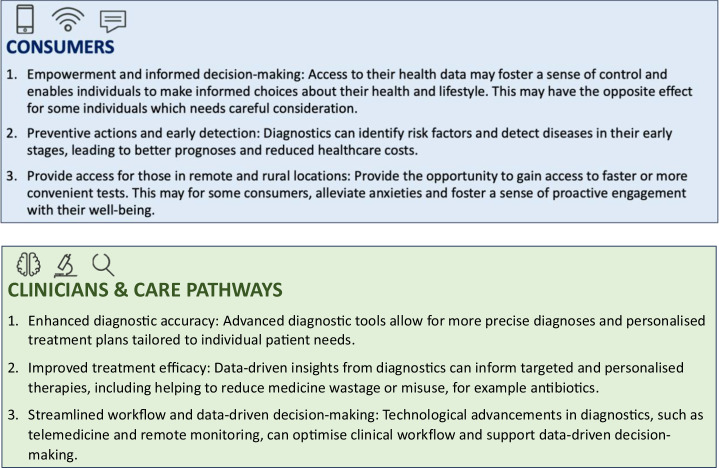
Exploring potential value for consumers, clinicians and care pathways. Parts of this figure utilises photos from following authors, all licensed under CC BY-SA https://www.stir.ac.uk/brand-bank/, https://thenounproject.com/icon/cognitive-3928976/, https://thenounproject.com/icon/time-series-2046072/, https://thenounproject.com/browse/icons/term/finger-print, Eye by Ian Anandara from https://thenounproject.com/browse/icons/term/eye, Doctor by SANB from https://thenounproject.com/browse/icons/term/doctor, Line Graph by Zach Bogart from https://thenounproject.com/browse/icons/term/line-graph.

Fuelled by advancements in wearable technology and direct-to-consumer testing, the market for consumer health continues to rise and gain momentum^13^. A potential key advantage of high-frequency sensing and measurement is the opportunity for more holistic and continuous measurement, avoiding traditional episodic assessment which may miss subtle or difficult-to-quantify impairments^6,9,14,15^. Whilst this has the potential to offer earlier detection and foster a more proactive health management approach for citizens and the public. Deployment and management of direct-to-consumer testing and consumer health technologies will require additional consideration to deliver equitable value for clinicians, consumers and care pathways^15–17^ (Fig. 2).

The role of diagnostics and the clinical workforce remain closely coupled, with clinicians playing a crucial role in navigating these challenges. Clinical judgement, supported by advancements in technology, may increasingly become more critical to act as a vital filter, interpreting test results within the context of a patient’s individual history, symptoms, and overall health picture. This clinician expertise may become increasingly prominent in the ways:

1. **Manage false positives and misinterpretations:** Alongside advancements in AI, by applying their knowledge of disease presentation, broader evidence base and test limitations, clinicians can help to differentiate true positives from misleading results.
2. **Weigh up potential benefits and negatives of interventions:** Clinicians can continue to engage in discussion with patients to weigh up the potential situational benefits and risks of treatment against the potential impact of different interventions, with the patients’ individual perspectives and situations in mind.
3. **Promote responsible data and resource utilisation:** Clinicians can help guide the patient and consumer in using and sharing their data effectively, focusing on tangible insights and clinically meaningful results for their own circumstances. This is increasingly important with broader sustainability factors in healthcare^2^.

## Finding an appropriate balance to realise diagnostics’ value

**Fig. 3:**
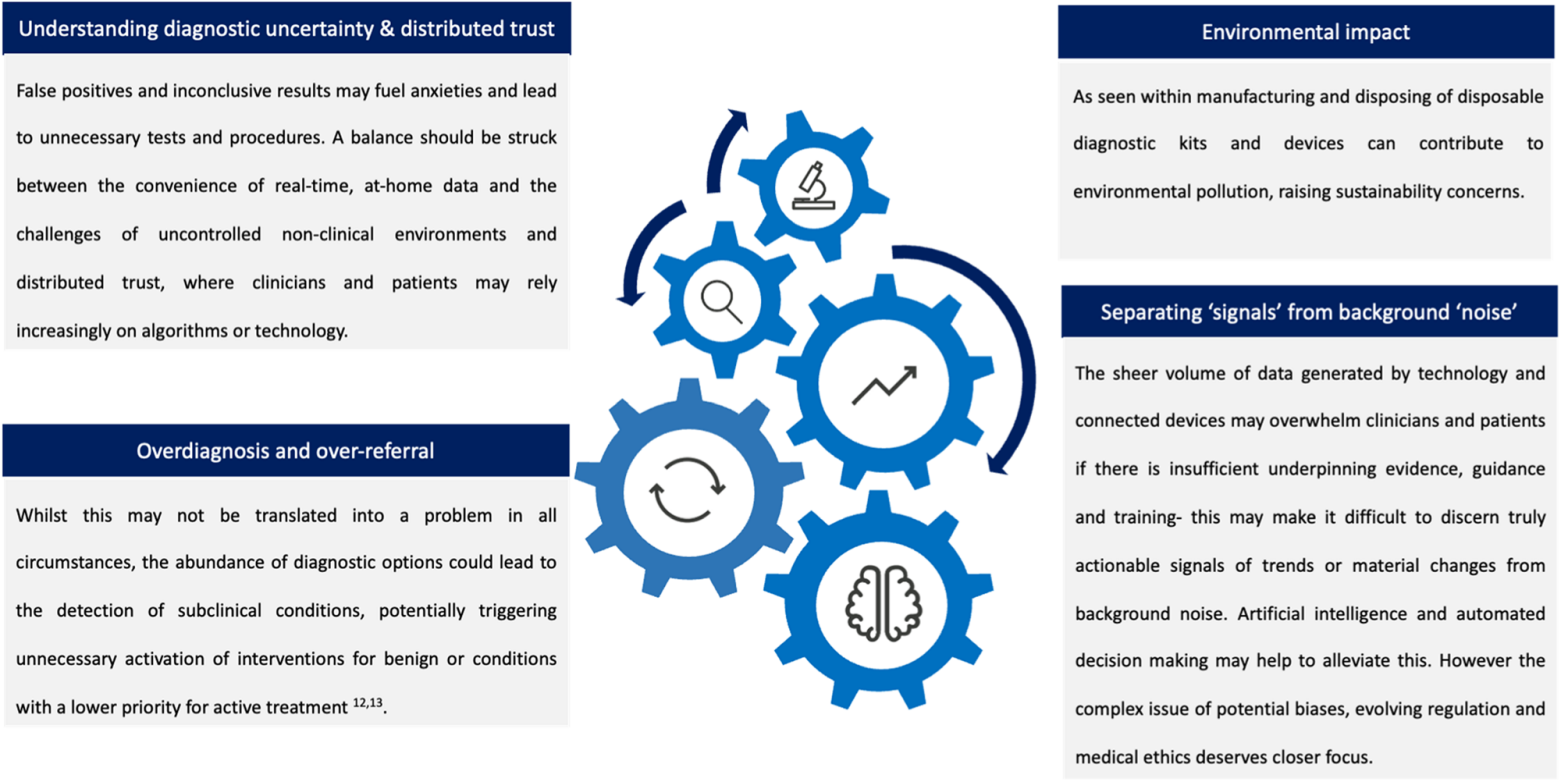
Challenges of diagnostics for the ecosystem. Parts of this figure utilises photos from the following authors, all licensed under CC BY-SA). https://www.stir.ac.uk/brand-bank/, https://thenounproject.com/icon/cognitive-3928976/, https://www.stir.ac.uk/brand-bank/, https://thenounproject.com/icon/cognitive-3928976/, https://thenounproject.com/icon/time-series-2046072/, fingerprint by Mister Pixel https://thenounproject.com/browse/icons/term/finger-print, Eye by Ian Anandara from https://thenounproject.com/browse/icons/term/eye/.

The potential positive impacts of direct-to-consumer diagnostics are undeniable, but its full realisation hinges on addressing the inherent challenges and embracing responsible utilisation. Striking a balance between empowering individuals, highlighting the important role of clinicians and ensuring responsible data interpretation and use of evidence will be critical. All of which deserve a multi-pronged approach. Firstly, the evidence base for any proposed deployment of technology. This can facilitate frameworks, guidelines and education to better equip both clinicians and the consumer with the knowledge and skills to understand and interpret diagnostic or related data responsibly. Additionally recognising the importance of the clinician-patient interaction**:** exploring mechanisms to continue fostering open and transparent communication between clinicians and patients to address anxieties, discuss uncertainties, and guide responsible data interpretation. Lastly a renewed focus on both the regulation and ethical considerations that are fit for purpose for the clinician, consumer and care pathway would be beneficial. Following this establishing clear guidelines and ethical frameworks to govern a spectrum of activities from the development, deployment, and utilisation of diagnostic technologies to the use of AI, including data analysis, with impacts on environmental sustainability and responsible data practices^7^ (Fig. 3).

## Conclusion and next steps

**Fig. 4:**
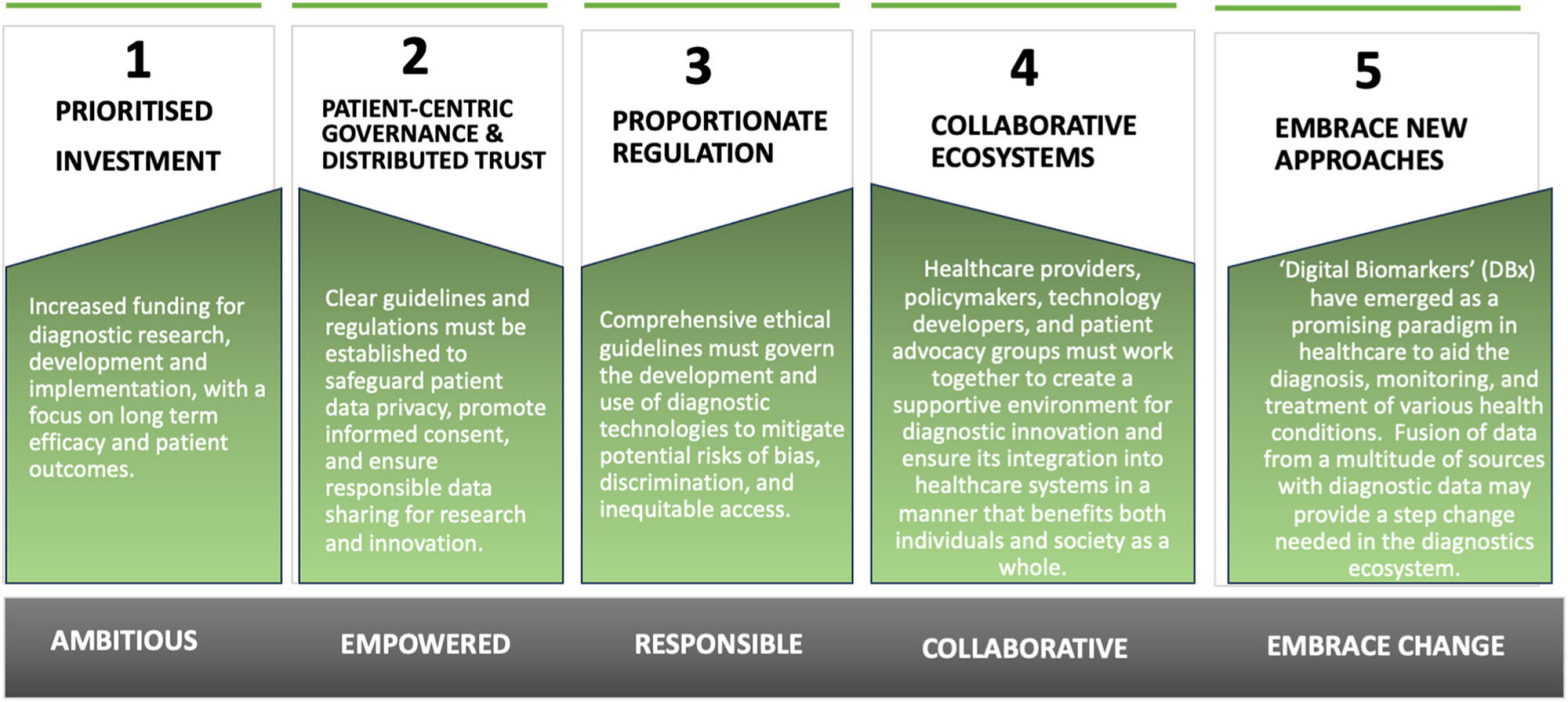
Harnessing the value of diagnostics may require a number of key steps. These include but not necessarily in this order, prioritising investment, patient dentric governance and disributed trust, proportionate regulation, colllaborative ecosystems, and an open mindset to adopting new approaches.

Novel diagnostic technologies and deployment stand poised to revolutionise health, but their full potential is likely only to be realised through proportionate regulation, sustained investment, and greater focus on distributed trust and patient empowerment. Despite influencing a vast majority of medical decisions, diagnostics currently receive a modest share of healthcare spending^5^. The perceived disconnect between ambition and impact could be further addressed via sustainable investment to ensure the widespread adoption and evaluation of impacts from diagnostic innovations. As previously discussed, the future of diagnostics will likely be in a more decentralised model, where patients play a proactive role in managing their health through connected technologies and new offerings through digital biomarkers and data-driven insights. However, this shift will necessitate a careful balance between innovation and ethical considerations surrounding data privacy, security, and equitable access. As outlined in Fig. 4, a multi-pronged approach may be a necessary precursor to harnessing value. Robust frameworks could be developed to protect patient rights and ensure that diagnostic advancements benefit all individuals, regardless of socioeconomic status or geographical location^18^.

By embracing these strategies, diagnostics may illuminate a brighter future for healthcare, where early detection, targeted treatments, and precision medicine lead to improved patient outcomes, reduced healthcare costs, and a more proactive approach to health management. While the exact path forward is not yet clear, the direction of travel is evident and it’s a critical time to harness the power of diagnostics to transform healthcare for the better.
